# Synthesis and host-guest chemistry of a thiophene-extended pillar[6]arene: toward applications in nitroaromatics removal and cell imaging

**DOI:** 10.3389/fchem.2026.1799183

**Published:** 2026-03-26

**Authors:** Tingting Chen, Fengqin Wang

**Affiliations:** School of Mechanical Engineering, Nantong Institute of Technology, Nantong, Jiangsu, China

**Keywords:** cell imaging, host-guest chemistry, nitroaromatics sensing, pillar[6]arene, synthesis

## Abstract

Over the past decade, significant progress has been made in the study of pillar [n]arenes; however, the development of their analogs remains underexplored, offering new opportunities for the discovery of functional materials. In this work, a luminescent thiophene-extended pillar [6]arene (**TPExP6**) featuring a π-rich cavity was designed and synthesized from 2,5-dibromothiophene and (4-formylphenyl)boronic acid via a five-step procedure with an overall yield of approximately 9.8%. This molecule combines an extended π-conjugated cavity with strong intrinsic emission, enabling it not only to form stable host–guest complexes with nitrobenzene derivatives for the effective removal of persistent nitroaromatic pollutants from water but also to be applied in tumor cell imaging. This achievement substantially advances the application of macrocyclic compounds in environmental protection and biomedical materials.

## Introduction

Macrocyclic compounds, characterized by their distinctive geometric structures and intriguing physicochemical properties, have garnered significant research interest in recent decades (Cai et al., 2025; Tang et al., 2024; Wu et al., 2025). Their emergence has not only accelerated the development of host–guest systems but also established a cornerstone for the advancement of supramolecular chemistry (Chen et al., 2024; Dishi et al., 2024; Han et al., 2023; Hua et al., 2022). Supramolecular chemistry and host-guest complexation have been profoundly advanced over the past decades, fueled by the design and development of a diverse range of synthetic macrocyclic hosts, among which crown ethers (Fang et al., 2019), cyclodextrins (Achkar et al., 2020), calixarenes (Nag and Rao, 2022), cucurbiturils (Liu et al., 2021) and pillar [n]arenes (Dai et al., 2025; Kato et al., 2023), represent landmark examples. The advent of each novel class of such hosts has consistently addressed fundamental challenges in host-guest chemistry, thereby continuously expanding the frontiers and enriching the scope of the entire discipline (Chen et al., 2023; Wu et al., 2022). Pillar [n]arenes, as the most classic category of macrocyclic molecules, are not only easily synthesized and functionalized but also possess diverse host-guest properties (Khalil-Cruz et al., 2021; Li M.-H. et al., 2021; Lu et al., 2022; Wada et al., 2023). Pillar [n]arenes have shown multiple applications, such as biomedicine imaging (Zhang et al., 2023), environmentally responsive materials (Chen et al., 2025; Li et al., 2023; Yu et al., 2023), catalysis (Wang, et al., 2023a; Zhou et al., 2024), and fluorescence materials (Yan et al., 2023; Yang et al., 2022; Zhu et al., 2023).

Supramolecular chemistry of pillar [n]arenes have been well investigated (Guo et al., 2021; Huang et al., 2020; Jiang et al., 2021; Li et al., 2020; Li W.-J. et al., 2021). However, pillar [n]arene analogs are not being exploited thoroughly. During the past 10 years, several novel pillararene derivatives with unique intrinsic structures, preorganized conformations, and attractive binding performances, such as biphen [n]arenes (Xu et al., 2022), leaning pillar [n]arenes (Wu et al., 2018), prism [n]arene (Yang and Jiang, 2020), geminiarene (Wu and Yang, 2019), fluoren [5]arenes (Wu et al., 2023) and so on, were reported. Despite these breakthroughs, there are still many bottlenecks to be addressed or strengthened. Firstly, pillar [n]arene-analogs with large cavity sizes usually have low yields. Secondly, macrocycles with extended π-conjugated and more electron-rich plane backbones are rarely reported (Wang et al., 2022a; Wang et al., 2022b; Wang et al., 2022c). Inspired by these, we assumed that merging the electron-rich thiophene unit into pillar [n]arene could generate a new class of macrocyclic hosts with extended cavity, extended π-conjugated plane and more electron-rich character. On the other hand, nitro-benzenes (NBs) play a crucial role as essential starting materials or intermediates in the synthesis of various compounds such as dyes, medicines, herbicidal agrochemicals, and rubber (Francisco et al., 2022; Khutorianskyi et al., 2016; Wu and Zhang, 2020). Due to the highly electronegative nature of fluorine and the electron-withdrawing properties of nitro-substituted groups, the degradation of NBs is extremely challenging. Consequently, the widespread use of NBs poses significant environmental risks and consequences (Zhao et al., 2023). Herein, we designed and synthesized an emissive thiophene extended pillar [6]arene (**TPExP6**) with a π-rich cavity. The pronounced sensitivity of the photophysical properties of **TPExP6** toward NBs, together with its capacity to interact with electron-deficient substrates, enables its application not only as a host material for forming host–guest complexes with NBs but also as an effective material for the removal of trace amounts of nitrobenzene from aqueous solutions. More importantly, due to **TPExP6**’s strong fluorescence emission properties, it can also be further utilized for live-cell imaging.

## Experiment section

### Synthesis of compound A1

A mixture of 2,5-dibromothiophene (0.48 g, 2 mmol), 4-formylphenylboronic acid (0.72 g, 4.80 mmol), K_2_CO_3_ (0.70 g, 5 mmol), and *N,N*-dimethylformamide (5 mL) was stirred under a N_2_ atmosphere for 30 min. Then, tetrakis (triphenylphosphine)palladium (0.12 g, 0.1 mmol) was added, and the reaction was stirred at 100 °C for 12 h. After completion, the mixture was allowed to cool naturally, and the resulting orange suspension was filtered and extracted with chloroform. The crude product was purified by column chromatography to afford **A1** as a brown solid (0.50 g, 84% yield). ^1^H NMR (400 MHz, CDCl_3_): 10.02 (s, 2H, CH), 7.93 (d, *J* = 8.3 Hz, 4H, ArH), 7.83–7.79 (m, 4H, ArH), 7.50 (s, 2H, Ha). ^13^C NMR (100 MHz, CDCl_3_): 190.32, 142.80, 138.40, 134.39, 134.13, 129.51, 125.27, 124.87.

### Synthesis of compound A2

Compound **A1** (1 g, 3.40 mmol) was dissolved in a mixed solvent of tetrahydrofuran and methanol (*V*_THF_: *V*_MeOH_ = 25 : 1; 52 mL). Sodium borohydride (1 g, 6 mmol) was added under ice-bath conditions. After reacting at room temperature for 30 min, deionized water was added to quench the reaction. The mixture was filtered and dried to afford a yellow solid, **A2** (0.9 g, 0.3 mmol), in a yield of 90%.^1^H NMR (400 MHz, DMSO-*d*_6_): 7.65 (d, *J* = 8.2, 4H, ArH), 7.51 (s, 2H, Ha), 7.37 (d, *J* = 8.2 Hz, 4H), 5.28 (s, 2H, -OH), 4.52 (s, 4H, CH_2_).

### Synthesis of compound A3

A suspension of compound **A2** (1 g, 3.3 mmol) in carbon tetrachloride (250 mL) was prepared. A solution of PCl_3_ (3 mL, 33 mmol) in carbon tetrachloride (10 mL) was added dropwise under an ice bath. The reaction was stirred at room temperature for 24 h. After completion, the mixture was washed with brine, saturated ammonium bicarbonate solution, and brine again. Finally, compound **A3** (0.56 g, 1.7 mmol) was obtained by column chromatography with a yield of 50%. ^1^H NMR (400 MHz, DMSO-*d*_6_): 7.72 (d, *J* = 8.2 Hz, 4H, ArH), 7.60 (s, 2H, Ha), 7.50 (d, *J* = 8.2 Hz, 4H, ArH), 4.80 (s, 4H, CH_2_).

### Synthesis of compound A4

Compound **A3** (1.1 g, 3.3 mmol) was dissolved in 20 mL of dichloromethane, followed by the addition of aluminum trichloride (0.9 g, 66 mmol). A solution of *p*-diethoxy benzene (4.6 g, 33 mmol) in dichloromethane (30 mL) was then added dropwise. The reaction was allowed to proceed at room temperature for 1 h, quenched with deionized water, and finally purified by column chromatography to yield compound A4 (880 mg, 1.6 mmol) with a yield of 50%. ^1^H NMR (400 MHz, CDCl_3_): 7.68 (s, 4H), 7.64–7.60 (m, 4H), 7.43–7.36 (m, 2H), 7.05 (s, 1H), 6.91–6.75 (m, 5H), 4.15 (t, *J* = 6.0 Hz, 4H), 4.10 (t, *J* = 6.0 Hz, 4H), 4.06 (s, 2H), 1.43 (t, *J* = 8.0 Hz, 6H), 1.34 (t, *J* = 8.0 Hz, 6H); ^13^C NMR (100 MHz, CDCl_3_): 156.52, 152.15, 146.27, 137.88, 132.02, 129.24, 128.68, 126.27, 117.47, 115.33, 114.28, 65.36, 63.69, 34.95, 15.08, 14.71.

### Preparation of compound TPExP6

Compound **A4** (160 mg, 0.3 mmol) and paraformaldehyde (24 mg) were dissolved in dichloromethane (20 mL). Boron trifluoride diethyl etherate (20 mL) was added dropwise at room temperature. After reacting for 25 min, the reaction was quenched with saturated sodium bicarbonate solution (10 mL). Column chromatography separation yielded compound **TPExP6** (80 mg, 0.07 mmol) with a yield of 52%. ^1^H NMR (400 MHz, CDCl_3_): 7.74 (d, *J* = 4.0 Hz, 2H), 7.69–7.67 (m, 6H), 7.61–7.58 (m, 6H), 7.51 (t, *J* = 6.0 Hz, 3H), 7.41 (s, 3H), 7.02 (s, 2H), 7.01 (s, 2H), 6.85 (d, *J* = 6.0 Hz, 2H), 6.77 (d, *J* = 6.0 Hz, 2H), 4.08 (dd, *J* = 16.0, 8.0 Hz, 16H), 3.81 (s, 12H), 1.43 (t, *J* = 6.0 Hz, 24H). ^13^C NMR (100 MHz, CDCl_3_): 153.02, 152.93, 146.27, 137.88, 132.02, 130.61, 130.36, 128.68, 126.27, 123.26, 115.14, 114.86, 66.75, 34.95, 32.01, 14.53. HR-MS (ESI) Calcd. for C_70_H_64_O_8_S_2_Na [M + Na]^+^: 1231.52, found: 1231.46; C_70_H_64_O_8_S_2_K [M + K]^+^: 1247.49, found: 1247.51.

## Materials and methods

All reagents (2,5-dibromothiophene, 4-formylphenylboronic acid, 4-formylphenylboronic acid, p-diethoxy benzene, et al.) were commercially available and used as supplied without further purification. Solvents were either employed as purchased or dried according to procedures described in the literature.

^1^H NMR and ^13^C NMR spectra were recorded on a Bruker AVIII-400 MHz spectrometer. All NMR used tetramethylsilane (TMS) as the internal standard. Bruker Micro-TOF spectrometer was used to investigate the High-resolution Mass (ESI) of the compounds. Fluorescence spectra were recorded on a Hitachi F-7000-Fluorescence Spectrophotometer. Confocal images were acquired using an Olympus FLUOVIEWFV1000-confocal laser scanning unit mounted on an IX81 fixed stage upright microscope. Scanning electron microscopy (SEM) investigations were carried out on a JEOL6390LVinstrument. Single crystal X-ray data were obtained on a Bruker D8 X-ray single crystal Venture diffractometer using Cu Kα radiation (λ = 1.54184 Å). SAINT5.0 and SADABS S3 programs are used for the reduction and absorption correction of crystal data. The resolution and refinement of the crystal structure are obtained on the SHELXTL-97 software. Using the direct or Patterson methods, all non-hydrogen source coordinates are obtained by using the differential Fourier method and the least square method. Then the geometric method and the difference value are used. The hydrogen atom coordinates were obtained by Fourier method, and the crystal structure was obtained.

## Results and discussion

### Synthesis of TPExP6

**SCHEME 1:**
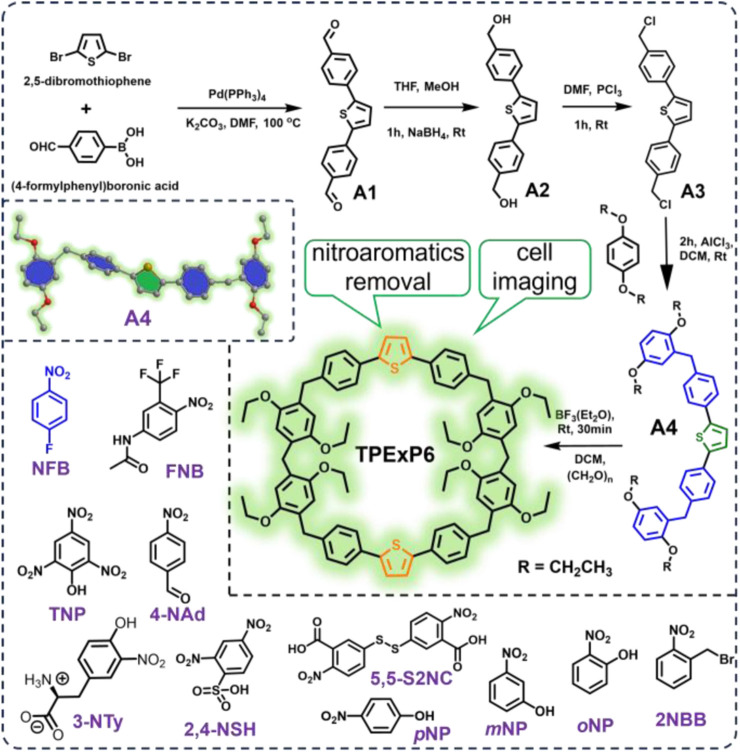
Synthetic route of thiophene extended pillar [6]arene (**TPExP6**), crystal structure of monomer **A4**, and chemical structures of nitrobenzene derivatives studied in this work.

Synthetic pathway of **TPExP6** is outlined in Scheme 1. The synthesis commenced with a C–C coupling between 2,5-dibromothiophene and (4-formylphenyl)boronic acid, followed by reduction of the resulting aldehyde group. The resultant intermediate was then treated with phosphorus trichloride (PCl_3_) to afford intermediate **A3**. In the subsequent step, **A3** was reacted with four equivalents of 1,4-diethoxybenzene in dichloromethane at room temperature, using aluminum trichloride (AlCl_3_) as a Lewis acid catalyst, leading to the formation of the hemicyclic derivative **A4**. Finally, **TPExP6** was obtained by treating **A4** with paraformaldehyde in dichloroethane under catalysis by boron trifluoride diethyl etherate (BF_3_·O(C_2_H_5_)_2_) at ambient temperature.

**FIGURE 1:**
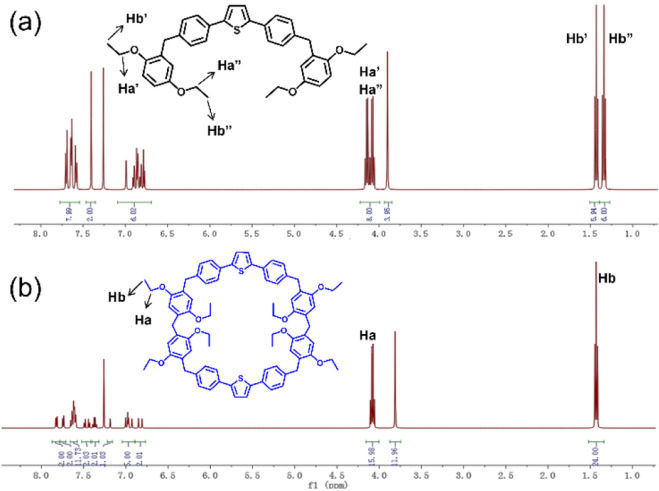
^1^H NMR spectra (400 MHz, CDCl_3_, 298 K) of **(a) A4** and **(b) TPExP6**. Ha and Hb were the protons of -CH_2_- and -CH_3_- on **TPExP6**, Ha’ and Ha” are the protons of -CH_2_- on **A4**, Hb’ and Hb” are the protons of -CH_3_- on **A4**.

The crystal structure (Supplementary Figure S1) of the acyclic precursor **A4** clearly shows two 1,4-diethoxybenzene units linked via methylene bridges to both sides of a thiophene core. In these units, the OCH_2_CH_3_ groups reside at ortho and meta positions relative to the bridging methylene carbon, placing them in distinct chemical environments. This inequivalence is confirmed by proton NMR measurements (Figure 1a), showing the splitting of the OCH_2_ and CH_3_ protons into distinct sets of signals (Haʹ/Haʺ and Hbʹ/Hbʺ, respectively). Upon cyclization to form the macrocycle **TPExP6**, the molecular symmetry increases, resulting in the coalescence of these split signals for the OCH_2_CH_3_ protons. Concurrently, the integration ratio of OCH_2_ protons to the bridging CH_2_ protons changes from 2:1 in precursor **A4** to 4:3 in **TPExP6**. These ^1^H NMR observations collectively provide strong evidence for the successful formation of the cyclic structure **TPExP6**. Furthermore, the synthesis was unequivocally confirmed by ESI-TOF-MS analysis, where the spectrum of **TPExP6** (Supplementary Figure S4) exhibited 2 m/z peaks of 1231.46 and 1247.51, which can be assigned to [M + Na]^+^ and [M + K]^+^ adducts, respectively.

### Photophysical and self-assembly properties

**FIGURE 2:**
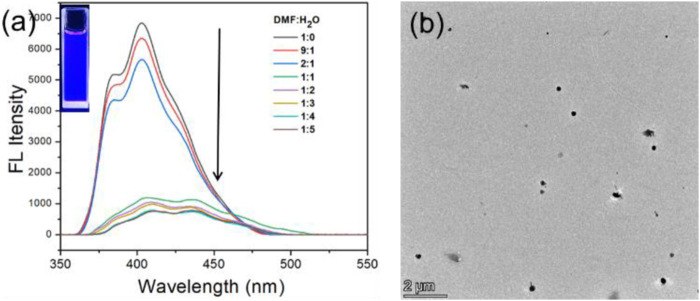
**(a)** Fluorescence spectra of **TPExP6** (1 μM) in different ratio of DMF/H_2_O. **(b)** TEM image of **TPExP6** aggregate in 50/50 DMF/H_2_O mixture.

Subsequently, the photophysical properties of **TPExP6** were investigated. UV/Vis spectrum of **TPExP6** in DMF exhibited an absorption maximum at around 330 nm (Supplementary Figure S5a). Fluorescence spectrum showed that **TPExP6** displayed bright blue emission centered at approximately 410 nm in DMF solution (Figure 2a). The fluorescence intensity is stable, and the quantum yield was calculated to be *Φ*_F_ = 28.2% (Supplementary Figures S5,S6; Supplementary Table S1). Upon gradual addition of water (a poor solvent) to the DMF solution, the fluorescence intensity decreased only slightly at water fractions below 30%. However, further increasing the water content led to a sharp decline in emission intensity, and when the water fraction exceeded 50%, the fluorescence was almost completely quenched (Figure 2a). This observation suggests that aggregate formation occurs at 50% water content, resulting in fluorescence quenching. Dynamic light scattering (DLS) data indicated the formation of **TPExP6** particles with a broad size distribution ranging from 100 to 500 nm in a 50/50 (*v*/*v*) water/DMF mixture (Supplementary Figure S8a), while transmission electron microscopy (TEM) imaging clearly revealed the presence of larger, uniformly sized nanoparticle aggregates (Figure 2b).

### Host-guest chemistry

**FIGURE 3:**
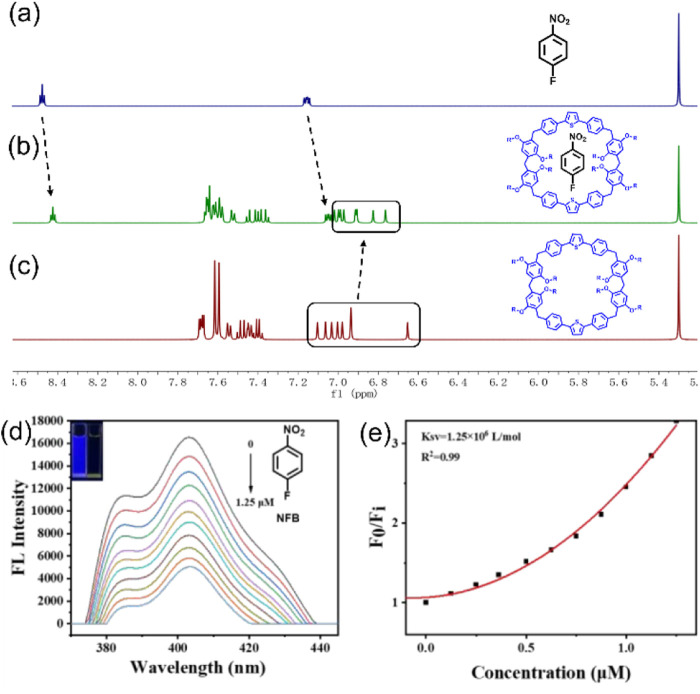
^1^H NMR spectra (400 MHz, CD_2_Cl_2_, 298 K): **(a)** 3 mmol NFB; **(b)** 3 mmol **TPExP6** and 3 mmol NFB; **(c)** 5 mmol **TPExP6**. **(d)** Fluorescence quenching of **TPExP6** (1 μM) at different NFB concentrations in DMF. **(e)** Stern–Volmer plot of **TPExP6** fluorescence quenching with NFB.

NBs pose severe risks to environmental safety and human health due to their high toxicity and persistence. Due to the electron-rich cavity of **TPExP6**, it can function as an excellent host to recognize host–guest complexation with NBs. Initially, the noncovalent interaction between **TPExP6** and a representative NB, 4-nitrofluorobenzene (NFB), using NMR characterizations. The NMR spectra (Figures 3a–c) revealed a noticeable upfield shift of the protons on NFB, which can be attributed to the shielding effect resulting from its encapsulation within the cavity of **TPExP6**. Subsequently, to gain further insight into the specific intermolecular forces between NFB and **TPExP6**, the quenching processes were monitored by emission intensity changes in response to NFB addition.

Figure 3d displays the gradual changes in the emission spectra of **TPExP6** (1 μM) upon incremental addition of NFB. A notable reduction in fluorescence intensity was observed as the concentration of NFB increased. To further understand the binding characteristics of the **TPExP6** NFB complex, the association constant (*K*sv) was evaluated using the Stern–Volmer relationship: F0/F = 1 - *K*_SV_ × [C], in which *F*0 (without the quencher) and F (with the quencher) are the fluorescence intensities and [C] denotes the concentration of NFB (Pérez-Márquez et al., 2022). The *K*_SV_ derived from this analysis was determined to be 1.25 × 10^6^ M^-1^, suggesting a strong noncovalent interaction between **TPExP6** and NFB (Figure 3e).

### Nitroaromatics removal

Employing an analogous experimental approach, we further investigated the host–guest interactions between **TPExP6** and a series of NBs (Supplementary Figures S9-S14), including 4-nitro-2-(trifluoromethyl)acetanilide (FNB), picric acid (TNP), 4-nitrobenzaldehyde (4-NBD), 3-nitrotyrosine (3-NTy), 2,4-dinitrobenzenesulfonic acid hydrate (2,4-NSH), 5,5'-disulfanediyl bis(2-nitrobenzoic acid) (5,5-S2NC), p-/m-/o-nitrophenol (*p*-NP, *m*-NP, *o*-NP), and 2-nitrobenzyl bromide (2NBB). As summarized in Supplementary Table S2, the binding constants (*K*sv) for **TPExP6** with these NBs were consistently greater than 10^3^ M^-1^, indicating robust complexation in all cases. These results demonstrate that **TPExP6**, as a macrocyclic host, exhibits broad applicability and effective binding generality toward diverse nitroaromatic substrates (Nagarkar et al., 2013).

**FIGURE 4:**
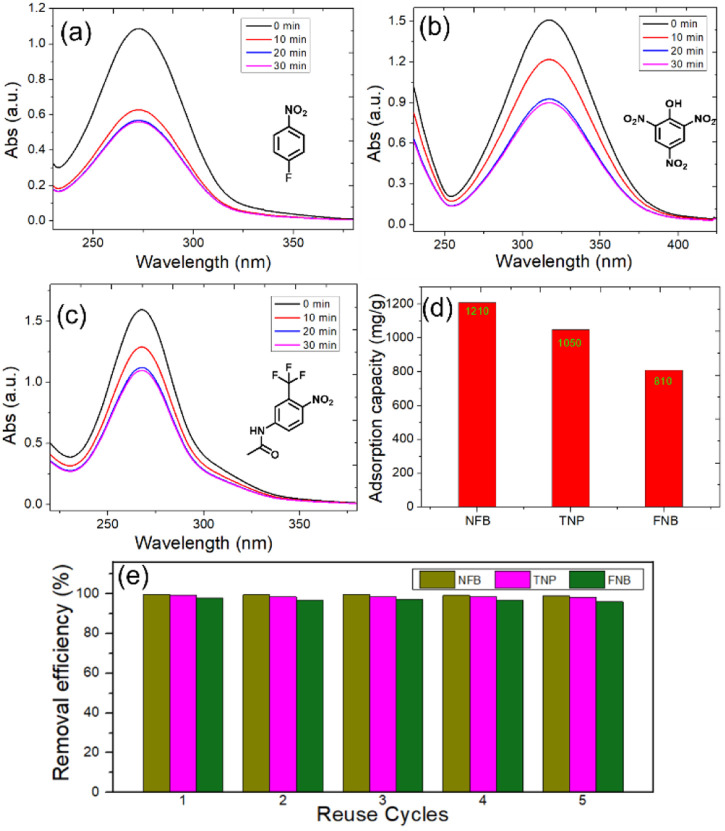
Uv-Vis spectra of 1.00 mg **TPExP6** submersed into aqueous solution of NBs (25 μg/mL, 100 mL): **(a)** NFB, **(b)** TNP, **(c)** FNB. **(d)** Adsorption capacity of **TPExP6** to NBs. **(e)** Removal efficiency of **TPExP6** to trace amounts of NBs against reuse cycles.

After confirming the host–guest complexation between **TPExP6** and NBs, we further investigated the application of **TPExP6** as a solid-phase material for removing trace-level NB pollutants from aqueous system. Firstly, the adsorption capacity of **TPExP6** toward NFB, TNP, and FNB was thus evaluated. We submersed 1.00 mg **TPExP6** into an aqueous solution of NBs (25 μg/mL, 100 mL). UV-Vis spectra were applied to monitor the pollutants removal efficiency. The peaks assigned to NBs decreased dramatically within the absorbance period, demonstrating the substantially decreased concentration of NBs (Figures 4a–c). The adsorption reaches the equilibrium in a short time (30 min), suggesting a rapid adsorption behavior. Quantitative analysis revealed that **TPExP6** absorbs NFB, TNP, and FNB with capacities of 1210, 1050, and 810 mg/g, respectively (Figure 4d). For the trace amounts of NBs in water systems (1 μg/mL), **TPExP6** achieved exceptional removal efficiencies of 99.9%, 99.2%, and 97.8% for these NBs. Furthermore, the removal efficiency was well maintained over five consecutive cycles without significant degradation (Figure 4e). These results collectively demonstrate the outstanding capability and reusability of **TPExP6** for removing trace NFB, TNP, and FNB from water.

### Cell imaging

**FIGURE 5:**
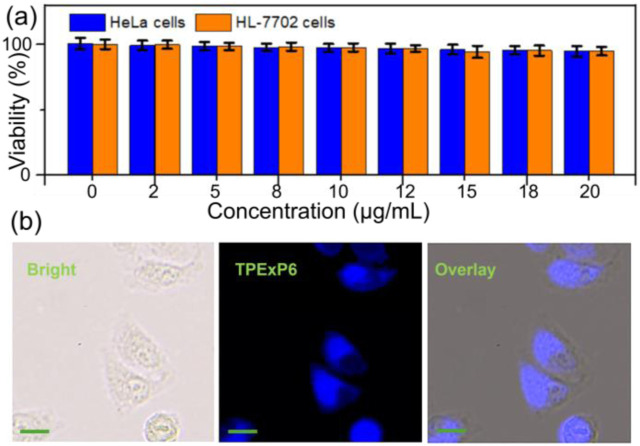
**(a)** Cell viabilities of HeLa and HL-7702 cells treated with **TPExP6** against different concentrations. **(b)** Confocal laser scanning microscopy images of HeLa cells cultivated with **TPExP6** for 4h. [C] = 20 μg/mL. Scale bar = 20 μm.

The strong fluorescence exhibited by **TPExP6** motivated us to explore its suitability as a biomaterial for live-cell imaging applications (Tang et al., 2023; Wang et al., 2022d; Wang et al., 2023b). First, cytotoxicities toward HeLa cells and HL-7702 cells were evaluated using the MTT (3-(4,5-dimethylthiazol-2-yl)-2,5-diphenyltetrazolium bromide) assay. Incubation with **TPExP6** across a concentration range of 1.00–20 μg/mL for 4 h resulted in no significant reduction in cell viabilities, demonstrating favorable biocompatibility and minimal cytotoxicity (Figure 5a). Subsequently, **TPExP6** was employed as a fluorescent probe for cellular imaging. Following a 4-h incubation period, confocal laser scanning microscopy (CLSM) observations revealed distinct intracellular blue fluorescence emission from **TPExP6**-treated HeLa cells, with the fluorescence primarily localized in the cytoplasm rather than the nucleus (Figure 5b). These results confirm that **TPExP6** can function as an effective fluorescent agent for live-cell visualization.

## Conclusion

In summary, a novel thiophene-extended pillar [6]arene (**TPExP6**) with electron-rich cavity and pronounced luminescent behavior was designed and prepared. This multifunctional macrocycle was efficiently constructed via a straightforward five-step synthetic route starting from commercially available precursors. The integration of an extended π-conjugated cavity with intense intrinsic emission endows **TPExP6** with the ability to function as an effective host for nitrobenzene derivatives, forming stable host–guest complexes in solution. Notably, these complexes are stabilized through charge-transfer interactions, which also underpin the capability of **TPExP6** to selectively capture and remove such recalcitrant nitroaromatic pollutants from aqueous media. More importantly, due to **TPExP6**’s strong fluorescence emission properties, it can also be further utilized for live-cell imaging. These findings highlight the considerable potential of emissive functionalized macrocycles as versatile and responsive platforms for advancing water purification technologies and cell imaging.

## Data Availability

The data presented in the study are deposited in the CCDC repository, accession number 2488938.
