# Supplementary material for: Synthesis and host-guest chemistry of a thiophene-extended pillar[6]arene: toward applications in nitroaromatics removal and cell imaging
Source: Front Chem. 2026 Mar 26;14:1799183. doi: 10.3389/fchem.2026.1799183 (PMC13062300; doi:10.3389/fchem.2026.1799183)
Supplement: Supplementary file 1 [file DataSheet1.docx]

Synthesis and host-guest chemistry of a thiophene-extended pillar[6]arene: toward applications in nitroaromatics removal and cell imaging

Tingting Chen* and Fengqin Wang

*School of Mechanical Engineering, Nantong Institute of Technology, Nantong, Jiangsu 226006, China*

**Supporting Information (16 pages)**

| 1 | **Materials and methods** | S2 |
| --- | --- | --- |
| 2 | **Synthesis of TPExP6** | S4 |
| 3 | **Fluorescence quantum yield** | S10 |
| 4 | **Self-assembly of TPExP6** | S11 |
| 5 | **Host-guest interaction** | S13 |

1. **Materials and methods**

**Materials**

All reagents were commercially available and used as supplied without further purification. Solvents were either employed as purchased or dried according to procedures described in the literature.

**Measurements**

^1^H and ^13^C NMR spectra were recorded on a Brucker AV400 spectrometer. UV/Vis spectra and the optical transmittance were recorded in a quartz cell (light path 10 mm) on a Shimadzu UV-3600 spectrophotometer equipped with a PTC-348WI temperature controller. Electrospray ionization mass spectra (ESI-MS) were measured by Agilent 6520 Q-TOF-MS. High-resolution Transmission electron microscopy (TEM) images were acquired using a Tecnai 20 high-resolution transmission electron microscope operating at an accelerating voltage of 80 keV. The sample for high-resolution TEM measurements was prepared by dropping the solution onto a copper grid. The grid was then air-dried. Solution samples were examined on a laser light scattering spectrometer (BI-200SM) equipped with a digital correlator (TurboCorr) at 636 nm at a scattering angle of 90°. The hydrodynamic diameter (Dh) was determined by DLS experiments at 25°C. Single crystal X-ray data were obtained on a Bruker D8 X-ray single crystal Venture diffractometer using Cu Kα radiation (λ = 1.54184 Å). SAINT5.0 and SADABS S3 programs are used for the reduction and absorption correction of crystal data. The resolution and refinement of the crystal structure are obtained on the SHELXTL-97 software. Using the direct or Patterson methods, all non-hydrogen source coordinates are obtained by using the differential Fourier method and the least square method. Then the geometric method and the difference value are used. The hydrogen atom coordinates were obtained by Fourier method, and the crystal structure was obtained. Confocal images were acquired using an Olympus FLUOVIEWFV1000-confocal laser scanning unit mounted on an IX81 fixed stage upright microscope.

1. **Synthesis of TPExP6**

Scheme S1. Synthetic route to **TPExP6**

Synthesis of Compound **A1**:

A mixture of 2,5-dibromothiophene (0.48 g, 2 mmol), 4-formylphenylboronic acid (0.72 g, 4.80 mmol), K₂CO₃ (0.70 g, 5 mmol), and *N*,*N*-dimethylformamide (5 mL) was stirred under a N₂ atmosphere for 30 min. Then, tetrakis(triphenylphosphine)palladium (0.12 g, 0.1 mmol) was added, and the reaction was stirred at 100 °C for 12 h. After completion, the mixture was allowed to cool naturally, and the resulting orange suspension was filtered and extracted with chloroform. The crude product was purified by column chromatography to afford the target product as a brown solid (0.50 g, 84% yield). ^1^H NMR (400 MHz, CDCl_3_): 10.02 (s, 2H, CH), 7.93 (d, *J* = 8.3 Hz, 4H, ArH), 7.83–7.79 (m, 4H, ArH), 7.50 (s, 2H, Ha). ^13^C NMR (100 MHz, CDCl_3_): 190.32, 142.80, 138.40, 134.39, 134.13, 129.51, 125.27, 124.87.

Synthesis of Compound **A2**:

Compound **A1** (1 g, 3.40 mmol) was dissolved in a mixed solvent of tetrahydrofuran and methanol (*V*_THF_ : *V*_MeOH_ = 25 : 1; 52 mL). Sodium borohydride (1 g, 6 mmol) was added under ice-bath conditions. After reacting at room temperature for 30 minutes, deionized water was added to quench the reaction. The mixture was filtered and dried to afford a yellow solid, **A2** (0.9 g, 0.3 mmol), in a yield of 90%.^1^H NMR (400 MHz, DMSO-d_6_): 7.65 (d, *J* =8.2, 4H, ArH), 7.51 (s, 2H, Ha), 7.37 (d, *J* = 8.2 Hz, 4H), 5.28 (s, 2H, -OH), 4.52 (s, 4H, CH2).

Synthesis of Compound **A3**:

A suspension of compound **A2** (1 g, 3.3 mmol) in carbon tetrachloride (250 mL) was prepared. A solution of PCl₃ (3 mL, 33 mmol) in carbon tetrachloride (10 mL) was added dropwise under an ice bath. The reaction was stirred at room temperature for 24 h. After completion, the mixture was washed with brine, saturated ammonium bicarbonate solution, and brine again. Finally, compound **A3** (0.56 g, 1.7 mmol) was obtained by column chromatography with a yield of 50%.^1^H NMR (400 MHz, DMSO-*d*_6_): 7.72 (d, *J* = 8.2 Hz, 4H, ArH), 7.60 (s, 2H, Ha), 7.50 (d, *J* = 8.2 Hz, 4H, ArH), 4.80 (s, 4H, CH_2_).

**Synthesis of compound A4**: Compound **A3** (1.1 g, 3.3 mmol) was dissolved in 20 mL of dichloromethane, followed by the addition of aluminum trichloride (0.9 g, 66 mmol). A solution of *p*-diethoxy benzene (4.6 g, 33 mmol) in dichloromethane (30 mL) was then added dropwise. The reaction was allowed to proceed at room temperature for 1 h, quenched with deionized water, and finally purified by column chromatography to yield compound **A4** (880 mg, 1.6 mmol) with a yield of 50%. ^1^H NMR (400 MHz, CDCl_3_): 7.68 (s, 4H), 7.64-7.60 (m, 4H), 7.43-7.36 (m, 2H), 7.05 (s, 1H), 6.91-6.75 (m, 5H), 4.15 (t, *J* = 6.0 Hz, 4H), 4.10 (t, *J* = 6.0 Hz, 4H), 4.06 (s, 2H), 1.43 (t, *J* = 8.0 Hz, 6H), 1.34 (t, *J* = 8.0 Hz, 6H); ^13^C NMR (100 MHz, CDCl_3_): 156.52, 152.15, 146.27, 137.88, 132.02, 129.24, 128.68, 126.27, 117.47, 115.33, 114.28, 65.36, 63.69, 34.95, 15.08, 14.71.


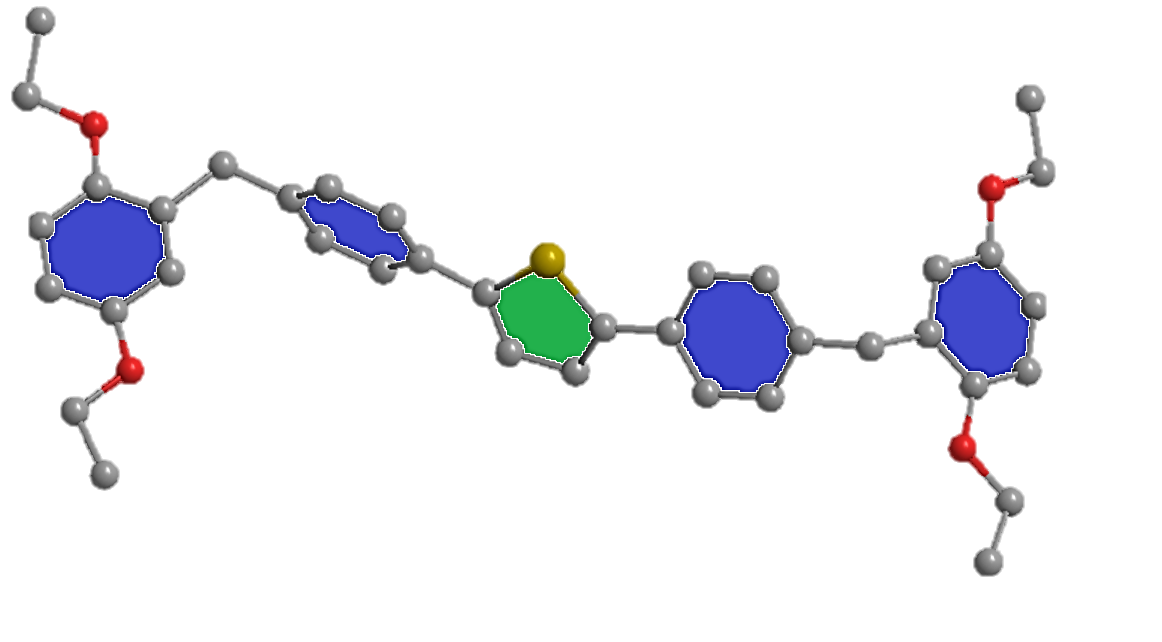


Fig. S1 The crystal structure of **A4**, H atoms were omitted for clarity.

**
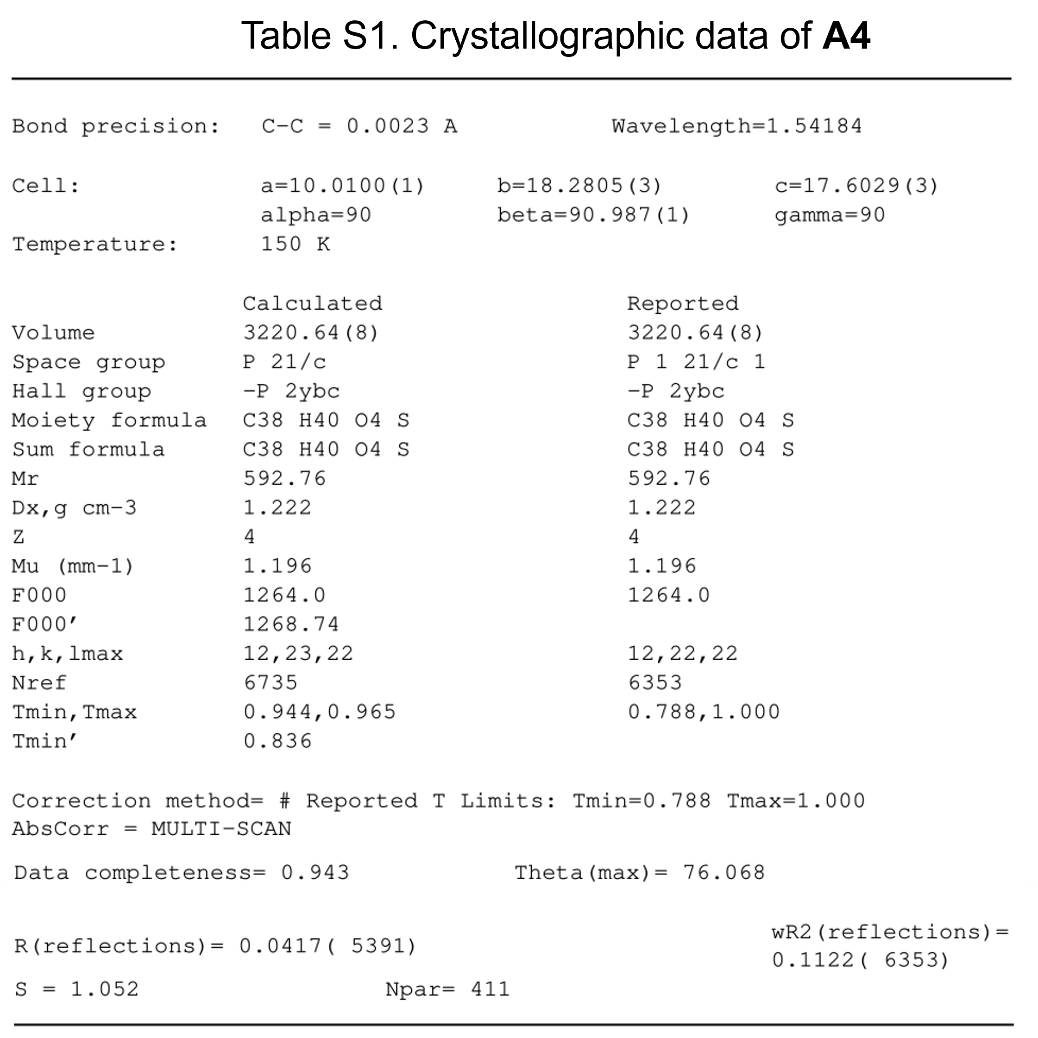
**

**Preparation of compound TPExP6**: Compound **A4** (160 mg, 0.3 mmol) and paraformaldehyde (24 mg) were dissolved in dichloromethane (20 mL). Boron trifluoride diethyl etherate (20 mL) was added dropwise at room temperature. After reacting for 25 minutes, the reaction was quenched with saturated sodium bicarbonate solution (10 mL). Column chromatography separation yielded compound **TPExP6** (80 mg, 0.07 mmol) with a yield of 52%. ^1^H NMR (400 MHz, CDCl_3_): 7.74 (d, *J* = 4.0 Hz, 2H), 7.69-7.67 (m, 6H), 7.61-7.58 (m, 6H), 7.51 (t, *J* = 6.0 Hz, 3H), 7.41 (s, 3H), 7.02 (s, 2H), 7.01 (s, 2H), 6.85 (d, *J* = 6.0 Hz, 2H), 6.77 (d, *J* = 6.0 Hz, 2H), 4.08 (dd, *J* = 16.0, 8.0 Hz, 16H), 3.81 (s, 12H), 1.43 (t, *J* = 6.0 Hz, 24H). ^13^C NMR (100 MHz, CDCl_3_): 153.02, 152.93, 146.27, 137.88, 132.02, 130.61, 130.36, 128.68, 126.27, 123.26, 115.14, 114.86, 66.75, 34.95, 32.01, 14.53. HRMS (ESI) Calcd. for C_70_H_64_O_8_S_2_Na [M + Na]^+^: 1231.52, found: 1231.46; C_70_H_64_O_8_S_2_K [M + K]^+^: 1247.49, found: 1247.51.


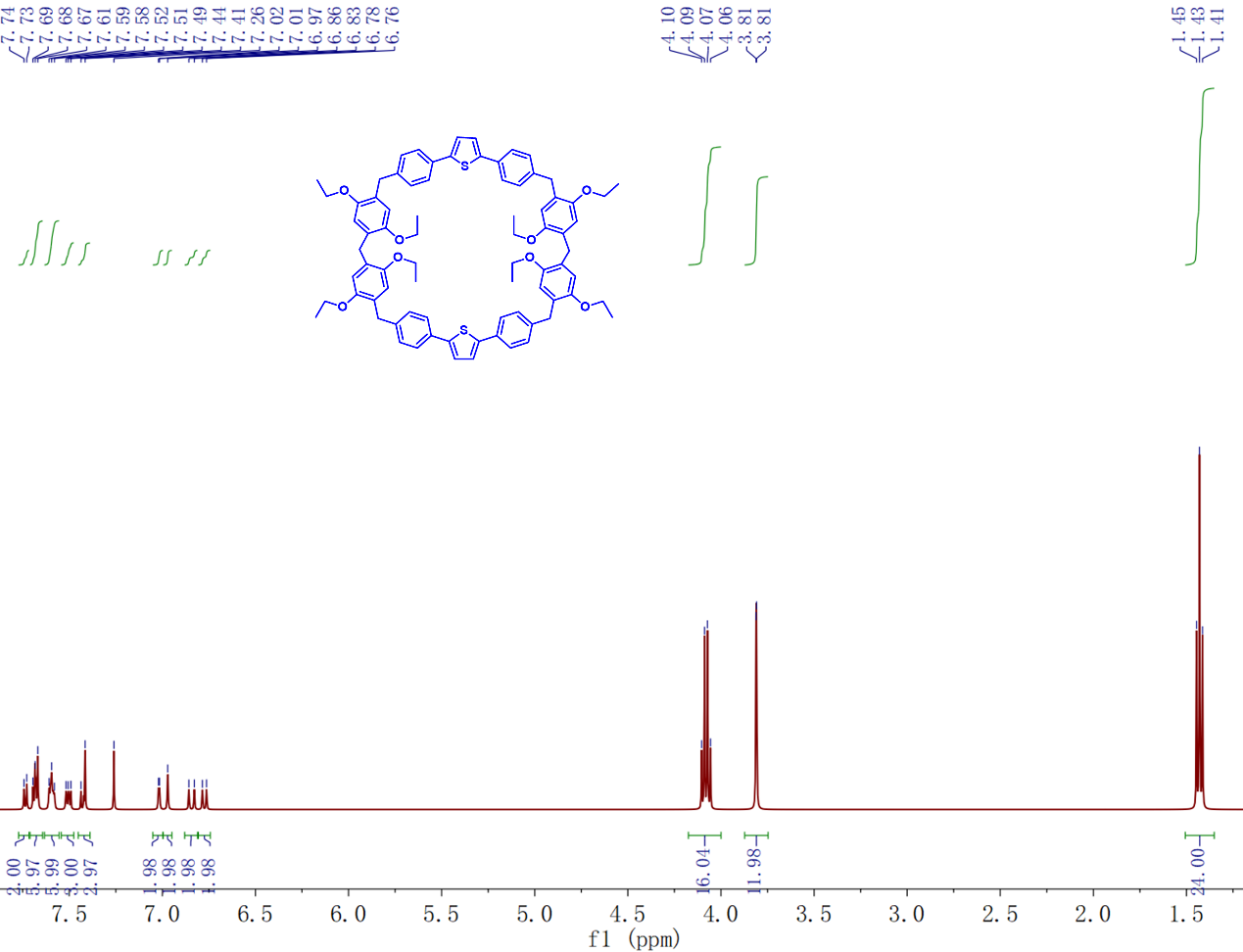


Fig. S2 ^1^H NMR spectra of **TPExP6** (400 MHz, CDCl_3_).


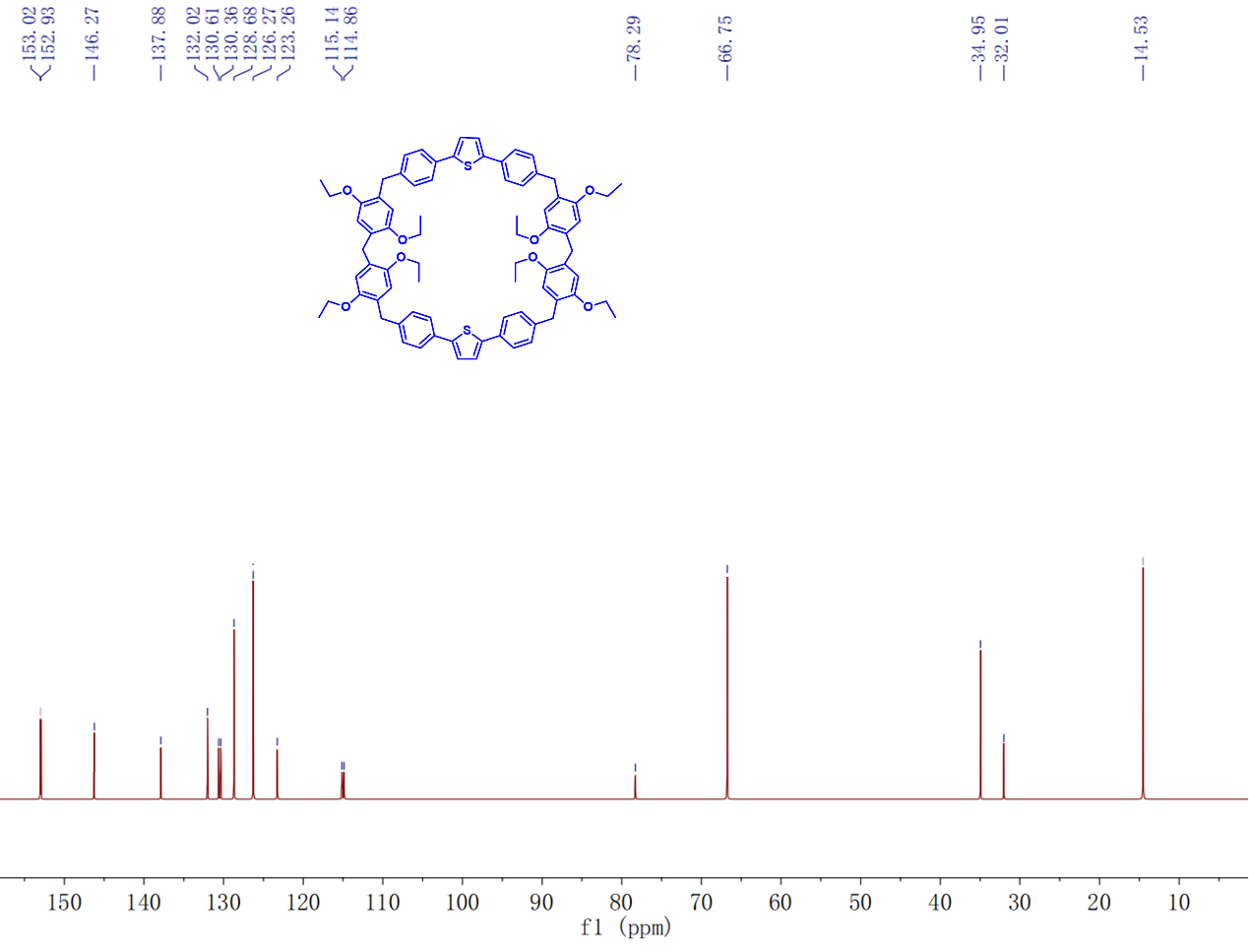


Fig. S3 ^13^C NMR spectra of **TPExP6** (101 MHz, CDCl_3_).


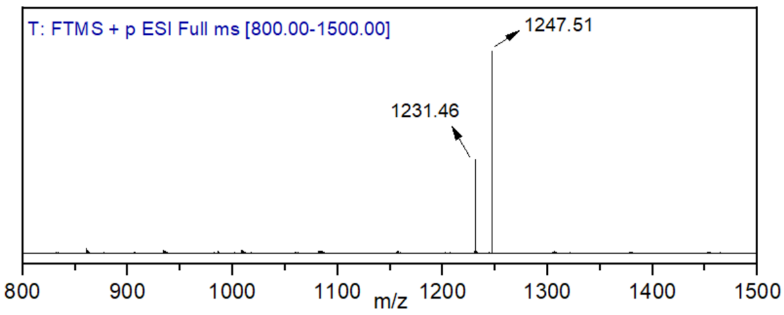


Fig. S4 MALDI-TOF-MS spectrum, MS (m/z): HRMS (ESI) Calcd. For C_70_H_64_O_8_S_2_Na [M + Na]^+^: 1231.52, found: 1231.46; C_70_H_64_O_8_S_2_K [M + K]^+^: 1247.49, found: 1247.51.

1. **Fluorescence Quantum Yield**

First, DMF solutions of the compound **TPExP6** and a standard solution of quinine sulfate (0.1 mol/L H_2_SO_4_) were prepared in sequence. The UV concentration standard curves of the compound **TPExP6** (Fig. S5) and quinine sulfate (Fig. S6) were initially measured. Subsequently, the fluorescence spectra of the quinine sulfate solution and the **TPExP6** solution were measured, and the integrated values of the fluorescence spectrum were recorded (Table S1). By calculating the relationship between the integrated values and the absorbance, the fluorescence quantum yield of **TPExP6** was obtained by substituting into formula ①.


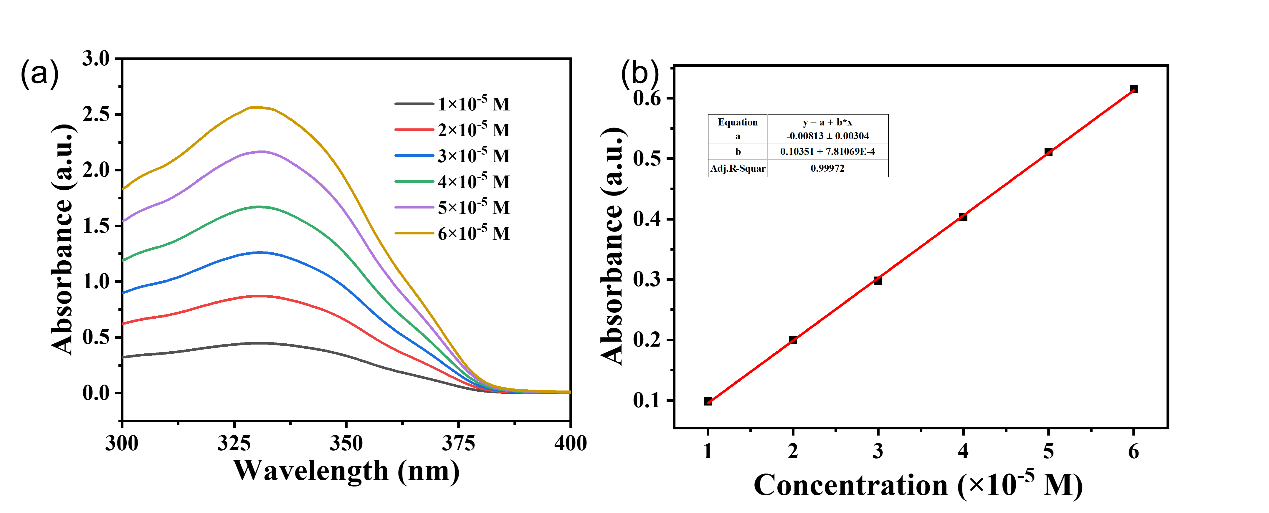


Fig. S5 (a) UV-Vis absorption spectra (DMF, r.t.) of different concentrations of **TPExP6**. (b) Absorbance intensity at 330 nm as a function of the concentration of **TPExP6**.


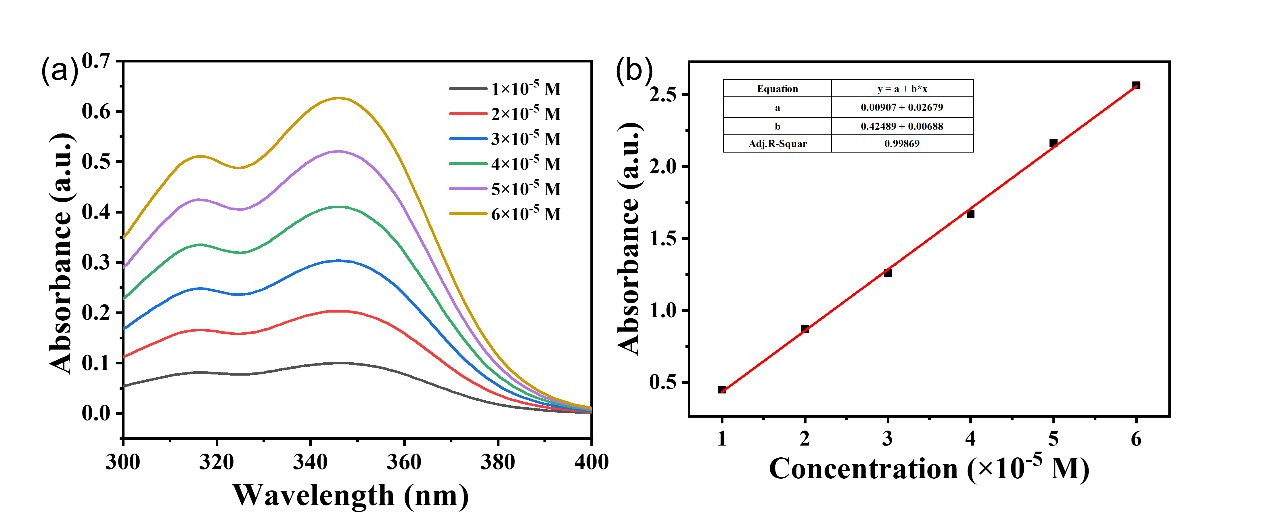


Fig. S6 (a) UV-Vis absorption spectra (0.1 mol/L H_2_SO_4_, r.t) of different concentrations of **Quinine Sulphate**. (b) Absorbance intensity at 350 nm as a function of the concentration of **Quinine Sulphate**.

Table S1 Absorbance and fluorescence integral area of dilute **quinine sulfate** solution and dilute **TPExP6** solution of standard reference

**quinine sulfate TPExP6**


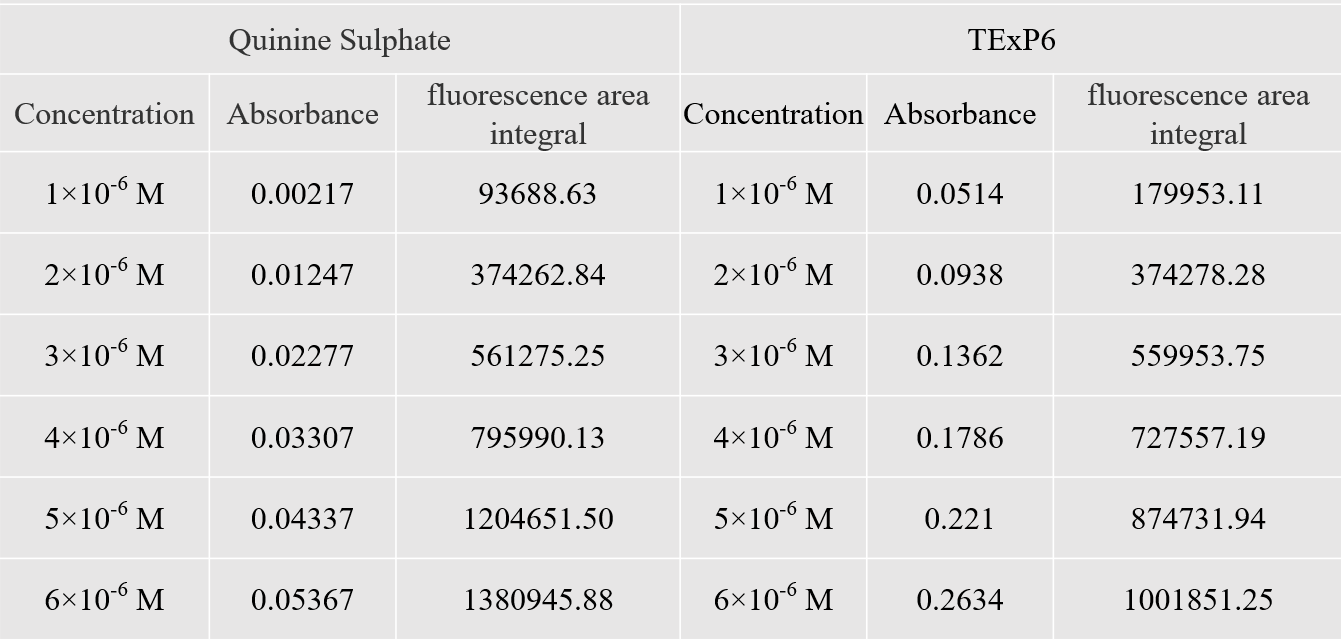


$\Phi_{X}$*=*$\Phi_{ST}$*(*$\frac{K_{X}}{K_{ST}}$*)(*$\frac{\eta_{X}^{2}}{\eta_{ST}^{2}}$*)* ①

$\Phi_{X}$= 0.28

1. **Self-assembly of TPExP6**

The fluorescence intensity of **TPExP6** was measured in mixed solutions of water and DMF at different ratios (Fig. S13a). It can be observed that water indeed affects the fluorescence emission capability of **TPExP6**, but the impact is negligible when only trace amounts of water are present (Fig. S13b).


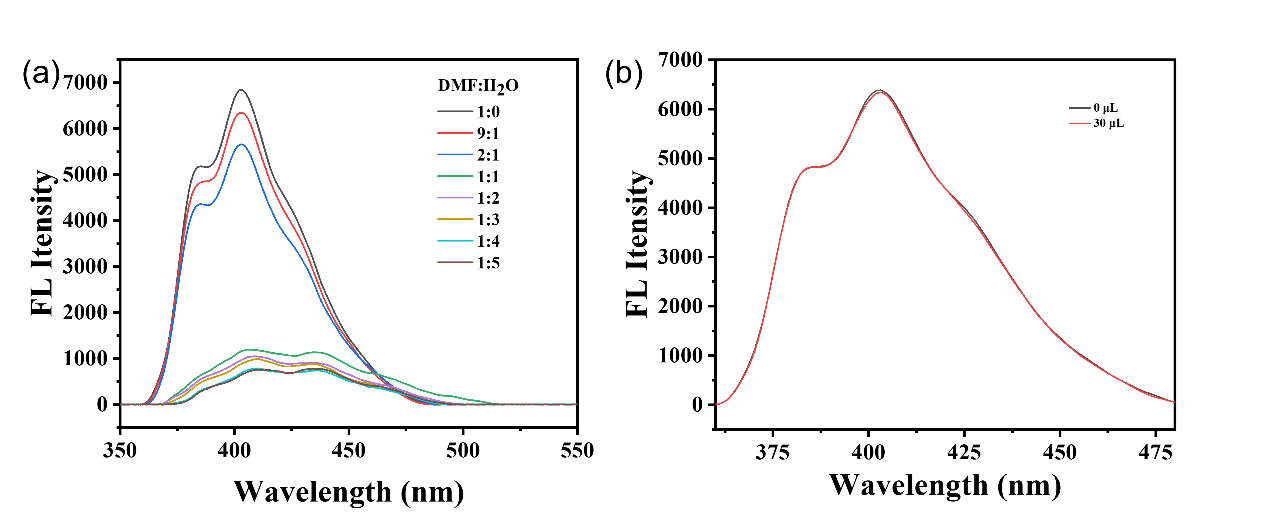


Fig. S7 (a) Fluorescence spectra of **TPExP6** in different ratio of DMF/H_2_O. (b) fluorescence spectra of **TPExP6** before and after adding water (30 μL ) to DMF (3 mL) solvent. [C] = 1.0 μM.


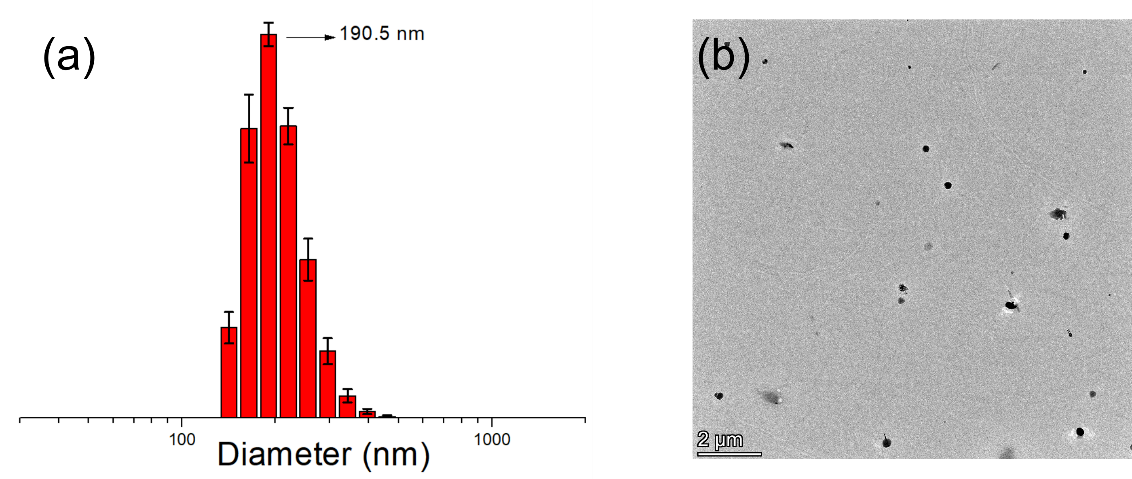


Fig. S8 (a) DLS and (b) TEM image of **TPExP6** aggregate in 50/50 DMF/H_2_O mixture.

1. **Host-guest interaction**

Subsequently, the host-guest interaction between **TPExP6** and some nitroaromatic compounds (NBs) was investigated by FL spectrum. The results (Figs. S15-S20) show the fluorescence spectra of **TPExP6** in DMF in the presence of different concentrations and types of NBs. Linear Stern-Volmer plots were constructed, and the quenching constants of different NBs for the DMF solution of **TPExP6** were calculated.


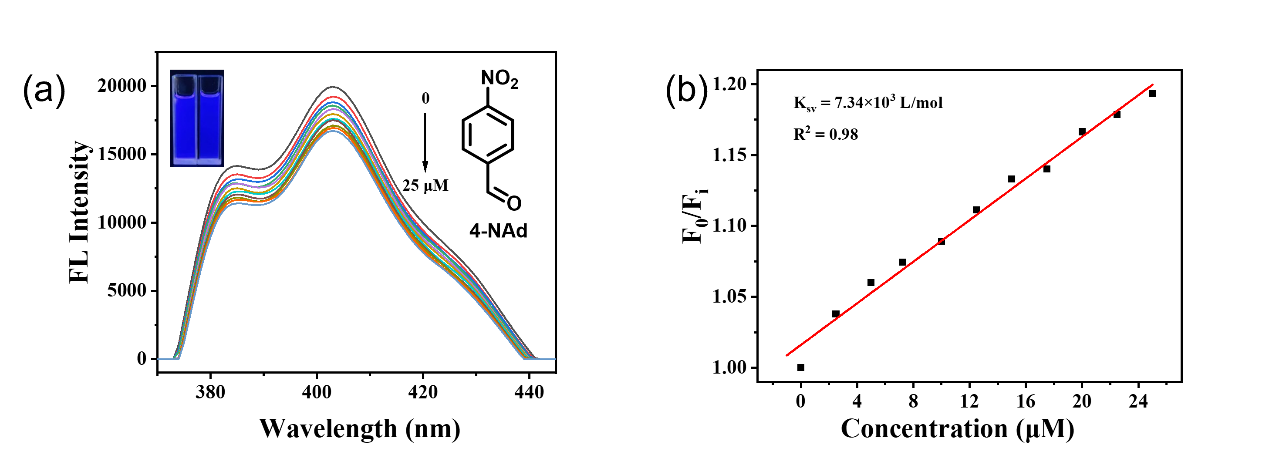


Fig. S9 (a) Fluorescence quenching of **TPExP6** (1 μM) at different **4-NAd** concentrations in DMF. (b) Stern–Volmer plot of fluorescence quenching with **4-NAd** (Ksv = 7.4×10^3^ M^-1^).


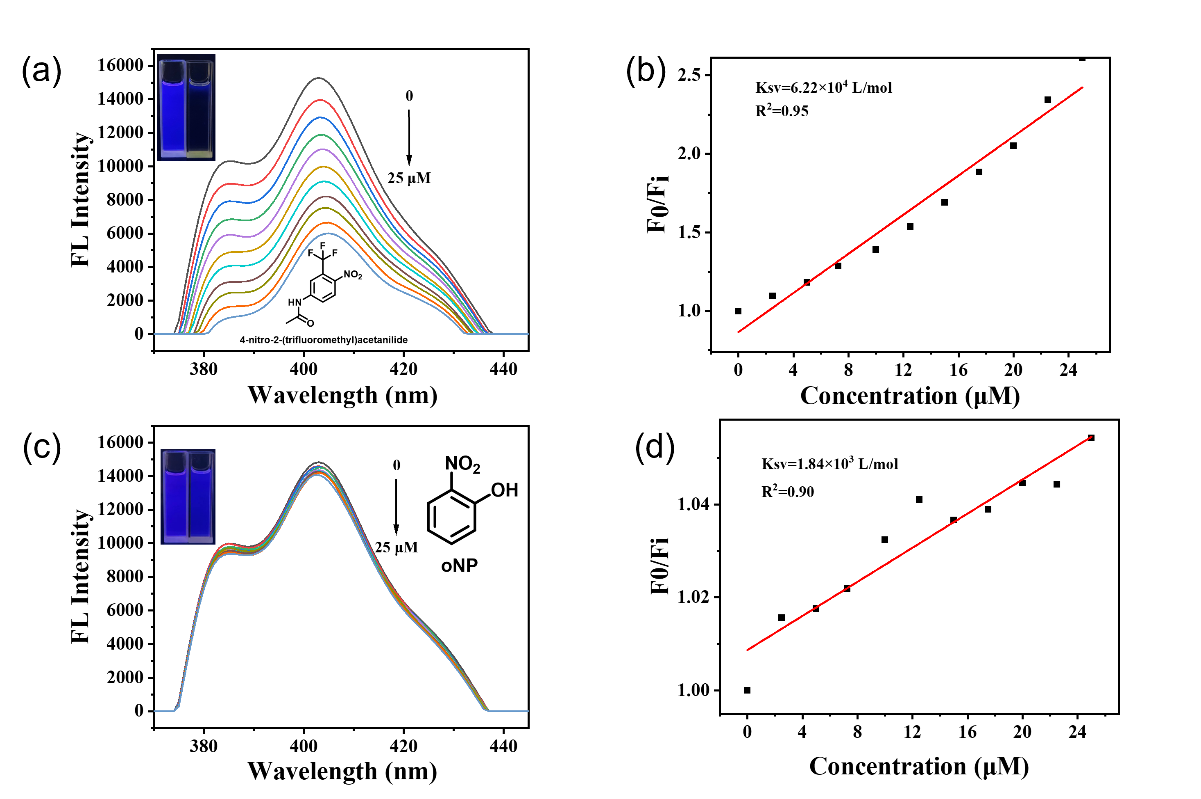


Fig. S10 (a) Fluorescence quenching of **TPExP6** (1 μM) at different 4-nitro-2-(trifluoromethyl)acetanilide **(FNB)** concentrations in DMF. (b) Stern–Volmer plot of A fluorescence quenching with **FNB** (*K*sv = 6.22 × 10^4^ M^-1^); (c) Fluorescence quenching of **TPExP6** (1 μM) at different **oNP** concentrations in DMF. (d) Stern–Volmer plot of A fluorescence quenching with **oNP** (*K*sv = 1.84 × 10^3^ M^-1^).


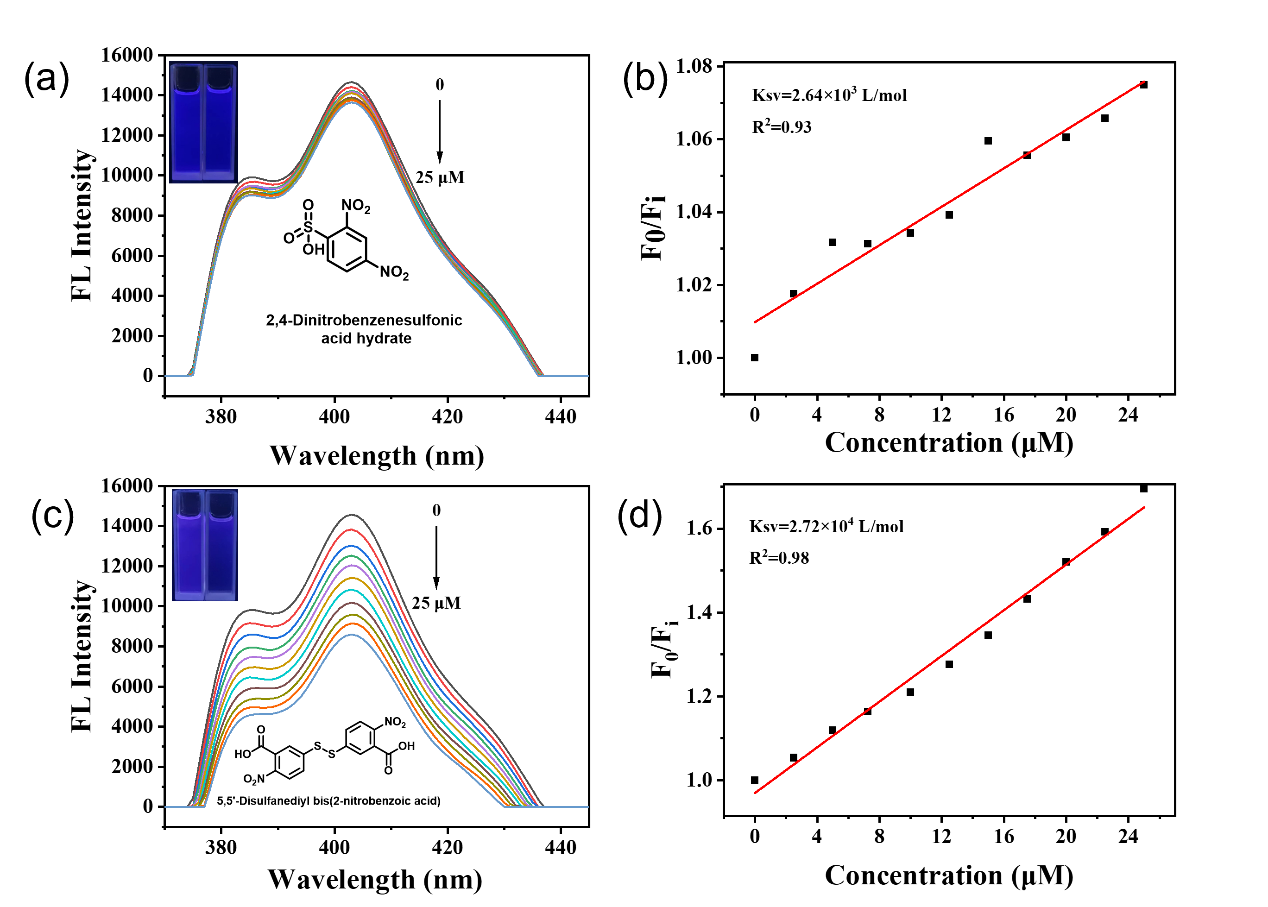


Fig. S11 (a) Fluorescence quenching of **TPExP6** (1 μM) at different 2,4-Dinitrobenzenesulfonic acid hydrate (**2,4-NSH**) concentrations in DMF. (b) Stern–Volmer plot of A fluorescence quenching with **2,4-NSH** (*K*sv = 2.64×10^3^ M^-1^); (c) Fluorescence quenching of **TPExP6** (1 μM) at different 5,5'-Disulfanediyl bis(2-nitrobenzoic acid) (**5,5-S2NC**) concentrations in DMF. (d) Stern–Volmer plot of A fluorescence quenching with **5,5-S2NC** (*K*sv=2.72 × 10^4^ M^-1^).


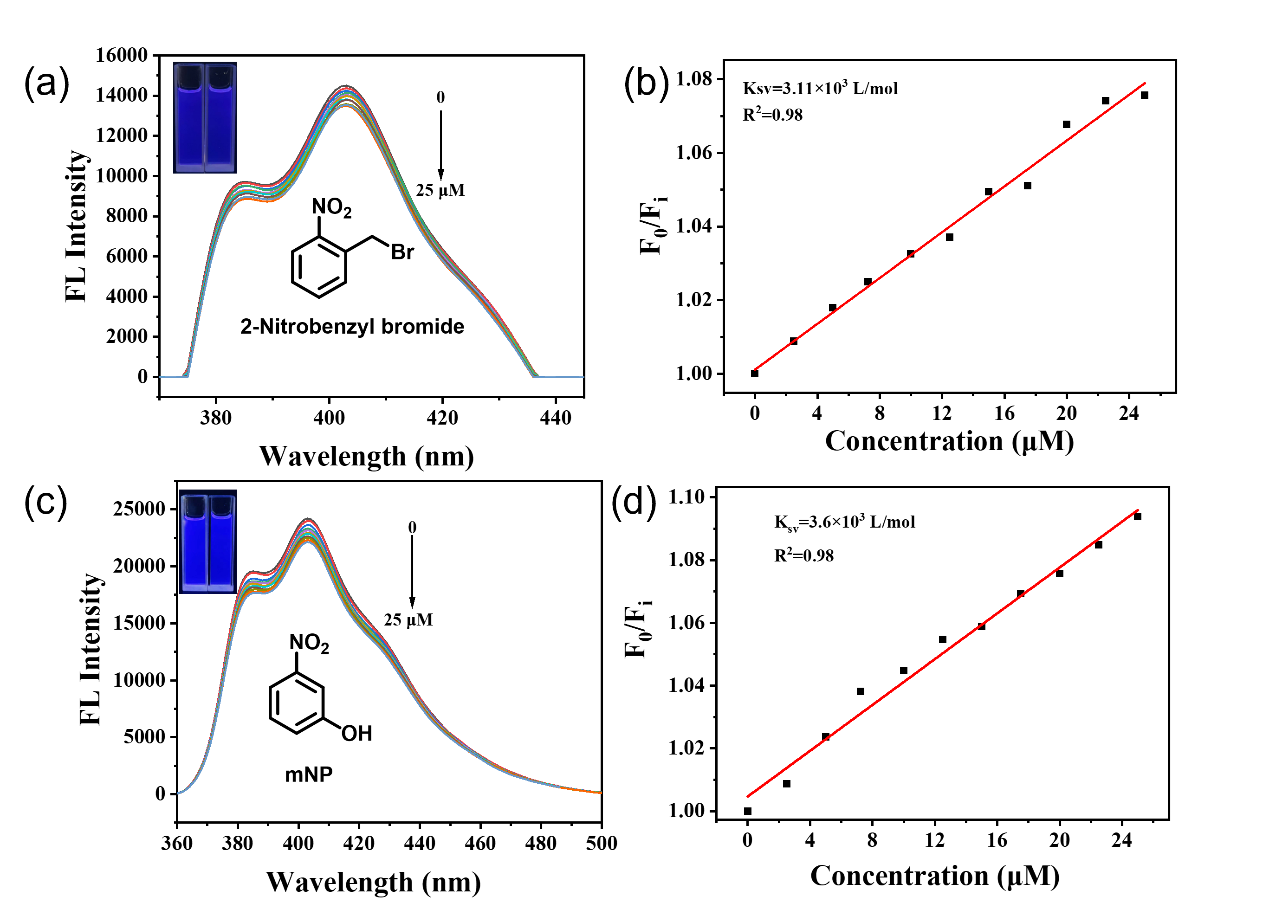


Fig. S12 (a) Fluorescence quenching of **TPExP6** (1 μM) at different 2-Nitrobenzyl bromide **(2NBB)** concentrations in DMF. (b) Stern–Volmer plot of A fluorescence quenching with **2NBB** (*K*sv = 3.11×10^3^ M^-1^); (c) Fluorescence quenching of **TPExP6** (1 μM) at different ***m*NP** concentrations in DMF. (d) Stern–Volmer plot of A fluorescence quenching with ***m*NP** (Ksv = 3.6×10^3^ M^-1^).


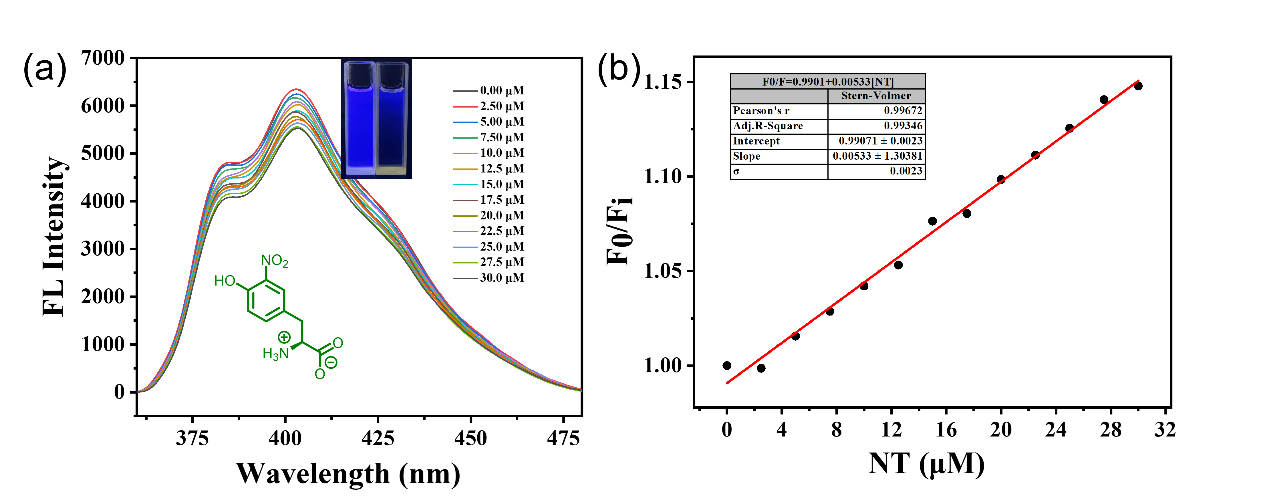


Fig. S13 (a) Fluorescence quenching of **TPExP6** (1 μM) at different 3**NTy** concentrations in 9 : 1 of DMF/H_2_O. (B) Stern–Volmer plot of **TPExP6** fluorescence quenching with 3**NTy** in 9 : 1 of THF/H_2_O (*K*sv=5.3 × 10^3^ M^-1^).


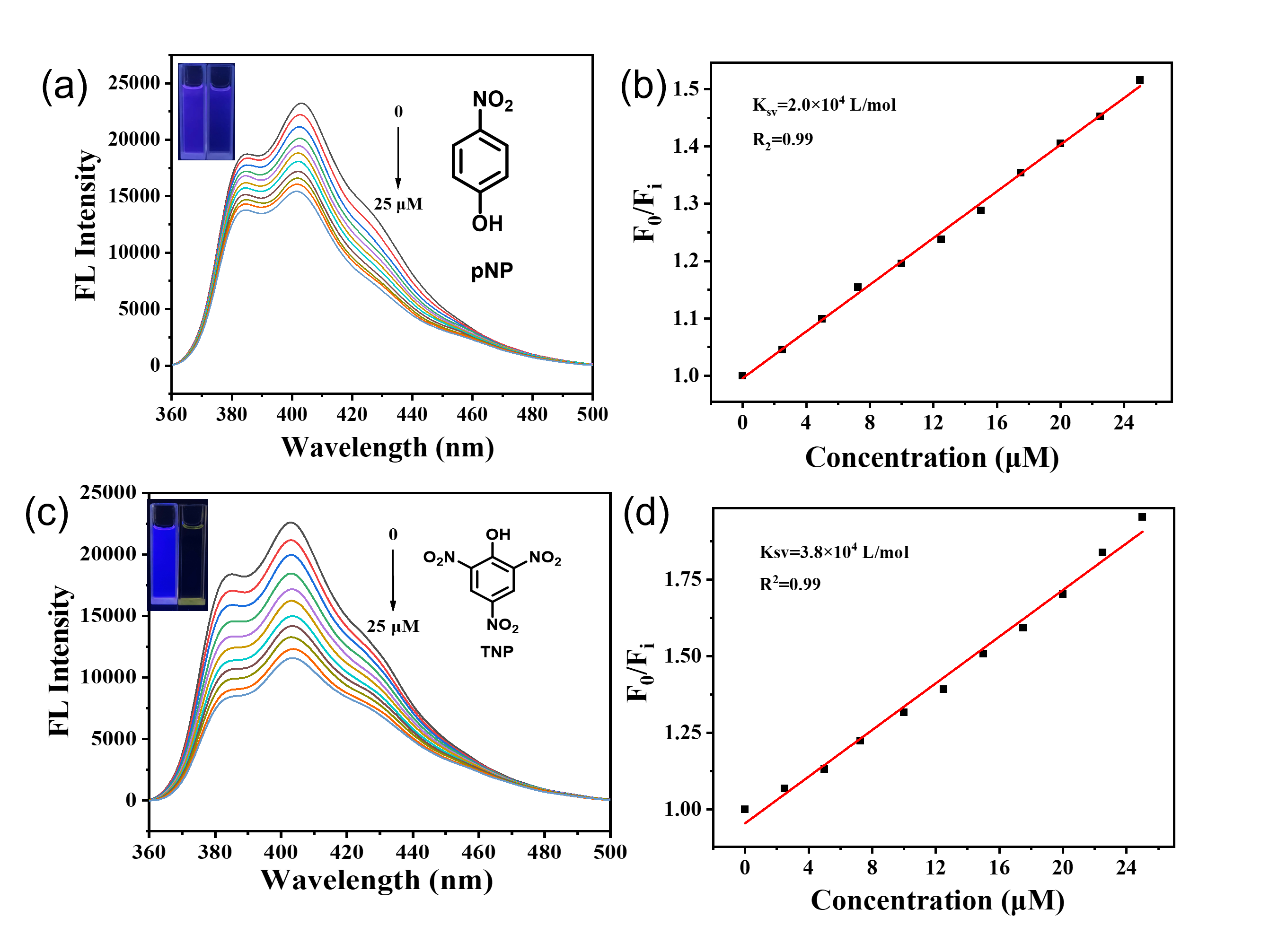


Fig. S14 (a) Fluorescence quenching of **TPExP6** (1 μM) at different ***p*NP** concentrations in DMF. (b) Stern–Volmer plot of A fluorescence quenching with ***p*NP** (*K*sv = 2.0×10^4^ M^-1^); (c) Fluorescence quenching of **TPExP6** (1 μM) at different **TNP** concentrations in DMF. (d) Stern–Volmer plot of A fluorescence quenching with **TNP** (*K*sv = 3.8×10^4^ M^-1^).

**Table 2**. *K*sv of NBs to **TPExP6**

| **NBs** | ***K*sv (M^-1^)** | **NBs** | ***K*sv (M^-1^)** |
| --- | --- | --- | --- |
| FNB | 6.22 × 10^4^ | 5,5-S2NC | 2.72 × 10^4^ |
| TNP | 3.80 × 10^4^ | *p-*NP | 2.01 × 10^4^ |
| 4-NAD | 7.34 × 10^3^ | *m-*NP | 3.60 × 10^3^ |
| 3-NTy | 5.33 × 10^3^ | *o-*NP | 1.84 × 10^3^ |
| 2,4-NSH | 2.64 × 10^3^ | 2NBB | 3.11 × 10^3^ |

**References**

S1. Chen T, Yin H, Chen Z, et al. *Small*, **2016**, 12(47): 6547-6552.

S2. Zheng B, Li Y, Tao F, et al. *Sens Actuators B-Chem.*, **2017**, 241: 357-363.
